# Physiology of Midkine and Its Potential Pathophysiological Role in COVID-19

**DOI:** 10.3389/fphys.2020.616552

**Published:** 2020-12-22

**Authors:** Giulia Sanino, Martino Bosco, Giuseppe Terrazzano

**Affiliations:** ^1^Farmacia Municipale 2, Azienda Sanitaria Locale (ASL) CN1, Fossano, Italy; ^2^Anatomia Patologica, Hospital “Michele e Pietro Ferrero”, Verduno, Italy; ^3^Department of Science, University of Basilicata, Potenza, Italy; ^4^Department of Translational Medical Sciences, University of Naples Federico II, Napoli, Italy

**Keywords:** midkine, SARS-CoV2, COVID-19, neutrophil infiltration, NETs, autophagy, immune responses

## Abstract

SARS-CoV2 infection not only causes abnormal severe pneumonia but also induces other relevant pathophysiological effects on several tissues and organs. In this regard, the clinical complications observed in COVID-19 include acute coronary syndrome, pulmonary thromboembolism, myocarditis and, in the severe cases, the occurrence of disseminated intravascular coagulation. Literature on COVID-19 highlighted the central role of the Renin Angiotensin Aldosterone System in the determinism of SARS-CoV2 cellular internalization in the target tissues. Lung degeneration and respiratory distress appear to be dependent on the perturbance of physiological mechanisms, such as the uncontrolled release of pro-inflammatory cytokines, a dysregulation of the fibrinolytic coagulative cascade and the hyperactivation of immune effector cells. In this mini review, we address the physiology of Midkine, a growth factor able to bind heparin, and its pathophysiological potential role in COVID-19 determinism. Midkine increases in many inflammatory and autoimmune conditions and correlates with several dysfunctional immune-inflammatory responses that appear to show similarities with the pathophysiological elicited by SARS-CoV2. Midkine, together with its receptor, could facilitate the virus entry, fostering its accumulation and increasing its affinity with Ace2 receptor. We also focus on Netosis, a particular mechanism of pathogen clearance exerted by neutrophils, which under certain pathological condition becomes dysfunctional and can cause tissue damage. Moreover, we highlight the mechanism of autophagy that the new coronavirus could try to escape in order to replicate itself, as well as on pulmonary fibrosis induced by hypoxia and on the release of cytokines and mediators of inflammation, correlating the interplay between Midkine and SARS-CoV2.

## Introduction

Severe acute respiratory syndrome coronavirus 2 (SARS-CoV2) infection not only causes abnormal severe pneumonia but also induces other relevant pathophysiological effects on several tissues and organs. In this regard, the cardiovascular complications observed in Corona Virus Disease of 2019 (COVID-19) include acute coronary syndrome, pulmonary thromboembolism, myocarditis and, in the severe cases, the occurrence of disseminated intravascular coagulation system (Vallamkondu et al., 2020; Verdecchia et al., 2020). Literature on COVID-19 highlighted the central role of the SAAR in SARS-CoV2 cellular internalization, particularly for the virus binding to angiotensin I converting enzyme 2 (ACE2) receptor expressed on the cell membrane of the tissues targeted by SARS-CoV2 (Hoffmann et al., 2020; Ingraham et al., 2020; Liu et al., 2020; Mycroft-West et al., 2020). Lung degeneration and respiratory distress appear to be dependent on the perturbance of host response mechanisms that could foster the uncontrolled release of pro-inflammatory cytokines, the dysregulation of the fibrinolytic coagulative cascade, as well as the hyper-activation of immune effector cells (Ackermann et al., 2020; Azkur et al., 2020; Becker, 2020; Stephen-Victor et al., 2020; Vallamkondu et al., 2020). Inflammation mediators, endothelial cells, neutrophils, and macrophages are responsible for the amplification of inflammation processes and concur to the cross talk between enzymatic cascades and signal pathways (Ackermann et al., 2020; Becker, 2020; Vallamkondu et al., 2020).

**Figure 1:**
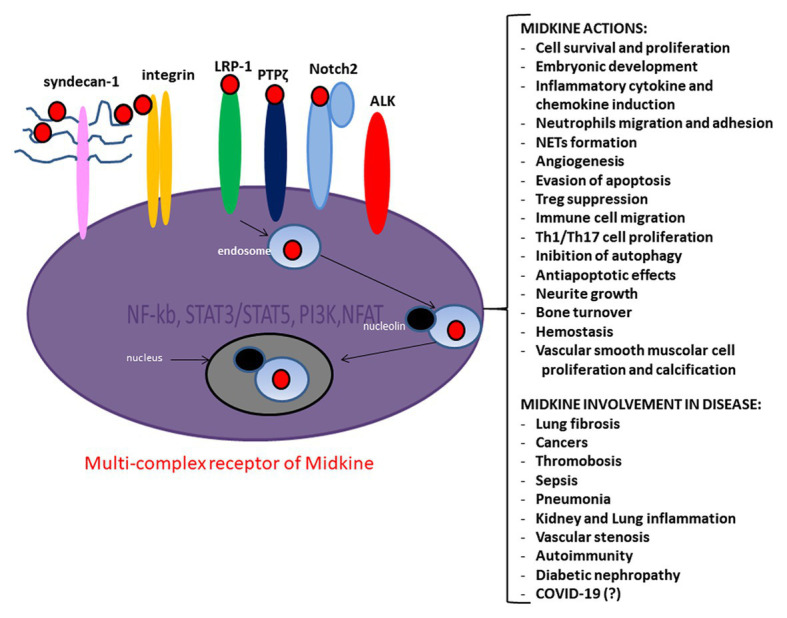
Midkine multi-complex receptor, actions and involvement in disease. Midkine multi-complex receptor includes Syndecan-n-1, low density lipoprotein receptor-related protein 1 (LRP-1), Neurogenic locus notch homolog protein 2 (Notch-2), integrins, protein tyrosine phosphatase zeta (PTP *ζ*), and Anaplastic lymphoma kinase (ALK). The signaling pathway of Midkine multi-complex receptor involves several molecules as the nuclear factor *kappa*-light-chain-enhancer of activated *B* cells (NF-kB), *signal transducer and activator of transcription 3 and 5 (*STAT3/STAT5), phosphatidylinositol 3-kinase (PI3K)/protein kinase B (AKT), and nuclear factor of activated T-cells (NFAT). Midkine promotes several actions ad is involved in various diseases.

Midkine is a growth factor able to bind heparin and showing a physiological role in embryonic development (Kadomatsu et al., 1988). Midkine is poorly expressed in the adult organism cells, while is highly incremented in cancer cells and correlated with a less favorable prognosis in cancer patients (O’Brien et al., 1996; Maeda et al., 2007). Midkine has a crucial role in the interplay between kidney and lung (Salvati et al., 2011), is involved in inflammation (Weckbach et al., 2011), angiogenesis (Weckbach et al., 2012), tumor growth (Kadomatsu, 2005), vascular stenosis (Weckbach et al., 2011), renal (Sato et al., 2001), neurodegenerative (Kadomatsu, 2005; Takeuchi, 2014), and autoimmune diseases (Takada et al., 1997; Kadomatsu, 2005; Figure 1). It is of note that Midkine is significantly involved in inflammation determinism (Weckbach et al., 2011), is induced during inflammation process, and enhances the recruitment of inflammatory cells (Kadomatsu et al., 2013; Figure 1). Midkine is expressed in several pathological renal conditions including diabetic nephropathy (Figure 1) and can exacerbate several kidney diseases through leukocyte recruitment (Weckbach et al., 2011). Patients with rheumatoid arthritis highly expressed Midkine (Weckbach et al., 2011). Endothelial lesions caused increase expression of Midkine that has been observed in macrophages infiltrated into the injured vascular wall (Weckbach et al., 2011).

Midkine can be easily detected by enzyme-linked immunosorbent assay (ELISA) in serum and urine (Ikematsu et al., 2000; Xia et al., 2016), and its tissue expression in histochemistry has been described (Kim et al., 2017).

Midkine is an important physiological mediator of Renin Angiotensin Aldosterone System (SAAR; Kadomatsu, 2010; Figure 1). SAAR regulates the migration and proliferation of smooth muscle cells and the extracellular matrix (ECM) production, the increased expression of adhesion proteins and pro-inflammatory cytokine production (Hoffmann et al., 2020; Ingraham et al., 2020; Liu et al., 2020). Plasma concentration of Midkine dramatically increased in patients with acute respiratory distress syndrome (ARDS; Zhang and Baker, 2017). Midkine appears to be overregulated upon mechanical stress in lung epithelial cells (Zhang et al., 2015; Zhang and Baker, 2017) and induces ACE2 level in the lung (Ezquerra et al., 2005; Kadomatsu, 2010). A recent study showed the interplay between Midkine and ACE2 in mechanically ventilated lung tissue (Huang S. et al., 2020). In addition, the overregulation of Midkine upon the mechanical stress was found in lung epithelial cells (Zhang et al., 2015; Zhang and Baker, 2017).

In this mini review, we focus the physiology of Midkine and its pathophysiological potential role in COVID-19, and we suggest to investigate Midkine as a putative biomarker of altered physiological conditions and/or a potential therapeutic target in the fight against pandemic COVID-19.

## Midkine, Heparan Sulfate, and Extracellular Matrix: A Role For Virus Entry Facilitation?

The ECM contains proteoglycans that are very important for the structural integrity and tissue morphogenesis and homeostasis (Frantz et al., 2010). Heparan sulfate proteoglycans (HSPGs) are mainly present in the ECM and in the cell cytoplasmatic membrane and bind the Heparan sulfate (HS) chains (Lu et al., 2011). Syndecans (SDC) are HSPGs acting as regulators of cell migration, endocytosis, and cell signals (Beauvais and Rapraeger, 2003; Afratis et al., 2012; Christianson and Belting, 2014; Gallagher, 2015; Changyaleket et al., 2017). HS chains, according to their different degree of sulfation, can interfere with the growth factors/receptors interplay and promote the signal activation (Lu et al., 2011; Changyaleket et al., 2017). ADAM and ADAMTS metalloproteases and heparanase (Lu et al., 2011; Changyaleket et al., 2017) shed “soluble syndecans,” which interact with the microenvironment, where they are released (Lu et al., 2011; Changyaleket et al., 2017). Several viruses use highly sulfated proteoglycans to bind the membrane surface of target cells (Rusnati et al., 2009; Cagno et al., 2019). The negative electrostatic proteoglycans charges interact with glycoproteins basic residues on the viral surface (Rusnati et al., 2009). The SARS-CoV2 spike protein (S-protein) interact with HS (Liu et al., 2020) and the binding affinity increases if HS is added to the S-protein proteolytic cleavage site (Liu et al., 2020). The HSPGs could increase the HCov-NL63 expression and could promote virus entry (Milewska et al., 2014; Kim et al., 2020).

Scientific Literature on COVID-19 highlighted the central role of the SAAR in the mechanisms of SARS-CoV2 cellular internalization, particularly for the occurrence of virus binding to ACE2 receptor expressed on the cell membrane of the tissues targeted by SARS-CoV2 infection (Hoffmann et al., 2020; Ingraham et al., 2020; Liu et al., 2020).

Midkine is a relevant component of heparin releasable endothelial proteins (HREPs) that are bound to the endothelial surface through proteoglycans and exert several specific functions in the vascular homeostasis (Novotny et al., 1993). Midkine strongly binds the hypersulfated structures of HS (Kaneda et al., 1996). Two Cardin and Weintraub (CW) motifs form a binding site based on heparan HS at the Midkine dimerization occurrence (Gallagher, 2015). The interaction with all three Midkine sulfate groups (6-O, 2-O, and n-sulfates) is crucial for the heparin-binding (Muramatsu et al., 1994; Kaneda et al., 1996; Asai et al., 1997; Maeda et al., 1999).

Midkine expression on cell surface strongly needs HS (Gallagher, 2015) and the tri-sulfate unit of HS is the binding site for Midkine itself (Kaneda et al., 1996). Midkine role as neuronal growth factor is impaired when cells are deprived of HS and activity is suppressed by heparin saccharides, which may block the site of interaction between HS and Midkine (Gallagher, 2015). The main receptor complex of Midkine includes Syndecan-1, glycosaminoglycans (GAGs), low density lipoprotein receptor-related protein 1 (LRP-1), Notch-2, integrins, protein tyrosine phosphatase *ζ* (PTP ζ), and anaplastic lymphoma kinase (ALK; Maeda et al., 1999). Other potential interplay between Midkine and some other extracellular ligands that bind Syndecans and/or interact with the LRP-1, as the tissue factor pathway inhibitor (TFPI), lipoprotein lypase, and several others, could have a relevant role in fostering Midkine activity and in determining other relevant biological functions (Kojima et al., 1996; Tinholt et al., 2015).

**Figure 2:**
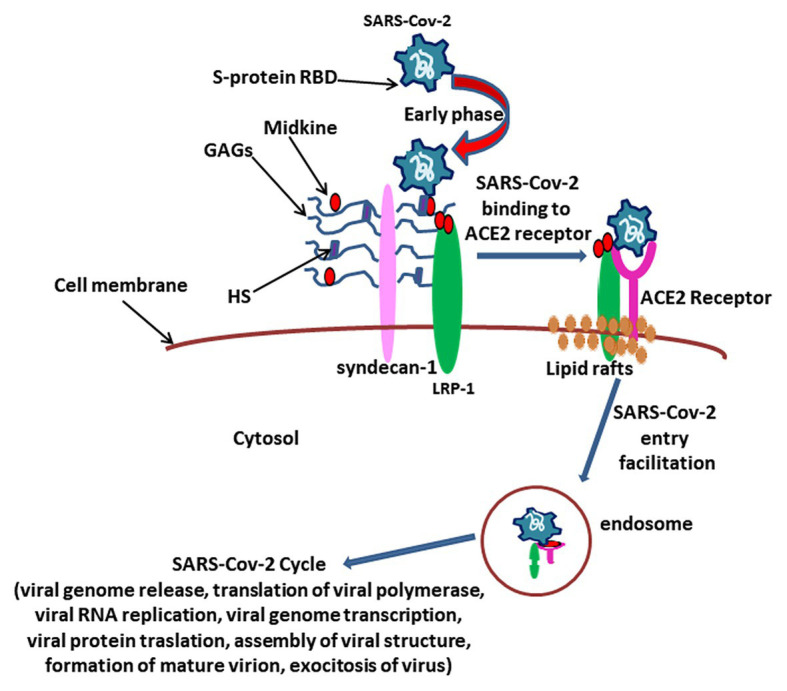
The hypothesis over the role for Midkine in SARS CoV2 viral attack. The complex between Midkine, Syndecan-1, glycosaminoglycans (GAGs), and Heparan sulfate (HS) could play a pivotal role in the early phase of virus attack by amplifying receptor-binding domain (RBD) sulfatation sites of Spike (S)-protein, in such way enhancing the Angiotensin I converting enzyme 2 (ACE2) receptor binding affinity and determining virus localization on the extracellular membrane. After SARS-CoV2/ACE2 receptor binding, Midkine could facilitate virus entry into the cell through LRP-1-mediated endocytosis, allowing the virus cycle as described (V’kovski et al., 2020).

We hypothesize that Midkine could be involved in the early stages of viral attack during COVID-19 (Figure 2). The S-protein fosters the entry of virus into cells (Hoffmann et al., 2020). The SARS CoV2 S-protein is composed by the S1 and S2 domains that are respectively correlated with the binding and fusion of virus to target cells (Hoffmann et al., 2020). The S1 expresses the receptor-binding domain (RBD) responsible for ACE2 receptor binding (He et al., 2004). S1 subunit of RBD exists in two different conformations, closed and open: the open RBD is able to bind the virus more than closed conformation (Hao et al., 2020). Enzymatic cleavage of protein S at the level of S1/S2 domains supports fusion of viruses to cell membranes *via* the S2 subunit (Liu et al., 2020). SARS-CoV2 S-protein interacts with both the cellular HS and ACE2 through its RBD and can simultaneously engage heparin and ACE2 (Clausen et al., 2020). Positively charged amino acids in a subdomain of RBD are responsible for the binding of heparin/HS complex *via* an interaction site that appears independent on the site involved in ACE2 binding (Clausen et al., 2020). SARS CoV2 protein S appears to bind HS cooperatively with ACE2 receptor on the cell surface (Clausen et al., 2020).

SARS-CoV2 may employ several different promoting factors to infect ACE2 receptor-expressing cells in the upper respiratory tract with greater efficiency than SARS-CoV, and this occurrence may explain the greater transmissibility of SARS-CoV2 compared to SARS-CoV (Hoffmann et al., 2020).

It is reasonable to assume that Midkine could amplify RBD sulfatation sites of S-protein, increasing the binding affinity with ACE2 receptor, and that Midkine would facilitate the open conformation of S1, in such way promoting the subsequent viral attack (Clausen et al., 2020).

## Midkine and Lipid Rafts

Scientific literature suggests the overall role of lipids in viral infection of target cells (Cervin and Anderson, 1991; Lajoie and Nabi, 2007; Li et al., 2007; Lu et al., 2008; Baglivo et al., 2020). Lipid rafts result in microdomains rich in cholesterol, glycosphingolipids, and phospholipids on the plasma membrane, potentially involved in the fusion, internalization, transport, and assembly of viral proteins of numerous viruses, including coronaviruses (Guo et al., 2017; Fecchi et al., 2020). Cholesterol represents the structural glue of lipid rafts (Fecchi et al., 2020). The ACE2 receptor is precisely located in the lipid rafts and is responsible for the initial phase of the viral infection of SARS-Cov2 (Fecchi et al., 2020). LRP-1 promotes endocytosis, is localized on lipid rafts, promotes the accumulation of cholesterol esters and the lipoproteins absorption (Actis Dato et al., 2020). Midkine is translocated into the nucleus by LRP-1 *via* nucleolin (Muramatsu et al., 2000).

In our hypothesis, the supposed interplay between the virus, Midkine, and HS and the presence of LRP-1 on lipid rafts might reveal new potential features of SARS-CoV2 infection mechanisms (Figure 2).

## Midkine and Immune Regulation: A Potential Role in Covid-19?

Immunological tolerance and immune homeostasis involve regulatory T cells (Tregs; Terrazzano et al., 2020). Tolerogenic dendritic cells (DCregs) influence the inducible Tregs development (Takeuchi, 2014). mTOR (mammalian target of rapamycin) is a protein kinase, involved in apoptosis, cell cycle, metabolic disorders and autoimmunity, carcinogenesis, inflammation and autophagy, immunoregulation, and tolerance (Terrazzano et al., 2020). mTOR forms two complexes: mTORC1 induces the T helper (Th) 1 and Th17 differentiation upon viral antigen presentation by dendritic cells (DC; Omarjee et al., 2020). mTORC2 mediates Th2 differentiation (Omarjee et al., 2020), while both complexes restrict Tregs differentiation. The two mTOR complexes are involved in the regulation of Tregs homeostasis (Omarjee et al., 2020). mTOR-dependent pathways may uncover molecular targets useful for controlling the cellular damage, oxidative stress, and hyperinflammation that occur in COVID-19. Recently, mTOR inhibition therapy has been hypothesized to mitigate the cytokine storm and to reduce hyperactivation of immune responses in COVID-19 (Terrazzano et al., 2020).

COVID-19 patients who undergo ARDS are characterized by highly enhanced pro-inflammatory cytokine production (the cytokine storm) and lung repair dysfunction, which is partially due to reduced or defective Tregs involvement (Gladstone et al., 2020). Midkine suppresses the generation DCregs, which drive the development of inducible Treg (Misa et al., 2017; Figure 1), and reduces phosphorylated STAT3 levels in DCregs (Misa et al., 2017). The specific inhibition of Midkine by RNA-based aptamer increased the DCregs and Tregs and decreased the autoreactive Th1 and Th17 cells, and it has been associated with the amelioration of the clinical symptoms in experimental autoimmune encephalomyelitis model (Takeuchi, 2014).

A dysregulation in the signaling pathways of mTOR, hypoxia-inducible factor 1 (HIF-1) alpha, tumor necrosis factor (TNF) has been identified during SARS-CoV2 infection (Appelberg et al., 2020). An increased expression of Midkine in the lung appears to be mediated by HIF-1 alpha (Reynolds et al., 2004). The respiratory epithelium responds to hypoxia through Midkine dependent HIF-1 alpha regulation (Reynolds et al., 2004). Midkine expression in human polymorphonuclear neutrophils (PMNs), monocytes, and endothelium increased by hypoxia (Weckbach et al., 2019).

Anaplastic lymphoma kinase (ALK) phosphorylates the insulin receptor substrate-1 and activates mitogen-activated protein (MAP) kinase and phosphoinositide 3 (PI3)-kinase leading to transcriptional activation of nuclear factor kappa-light-chain-enhancer of activated B cells (NF-*κ*B; Filippou et al., 2020). Filippou et al. (2020) recently reported that Midkine modulates the activity of the protein kinase B (Akt)/mTOR axis, *via* the ALK receptor, to prevent cell death mediated by cannabinoid-induced autophagy. Autophagy is a useful mechanism against viral infection. Autophagy plays a role in innate immunity, in the degradation of viruses or intracellular pathogens, and in the presentation of pathogens to the immune system (Fecchi et al., 2020). Viruses evolved mechanisms to escape the autophagic process (Carmona-Gutierrez et al., 2020).

SARS-CoV2, similarly to MERS-CoV, is able to reduce autophagy in infected cell lines by reducing the mTORC1-pathway, autophagy-related signaling, and the fusion between autophagosome and lysosome (Fecchi et al., 2020). SARS-CoV2 could benefit from reducing autophagy, preventing viral degradation, and improving the availability of double membrane vesicles (DMVs) needed for viral replication (Fecchi et al., 2020).

## Midkine a Key Factor For Neutrophil Activation in Covid-19?

The activation of neutrophils is very relevant during COVID-19 occurrence (Leppkes et al., 2020). In the course of inflammatory diseases, neutrophils excrete chromatin, histones and the contents of their own granules in a cellular process described as neutrophil extracellular trap (NET) formation (Leppkes et al., 2020). NET has been correlated to lung disease (Leppkes et al., 2020), neutrophils from pneumonia-associated ARDS undergo NET formation (Leppkes et al., 2020), extracellular histones are elevated in ARDS (Lv et al., 2017), and NET process is described in COVID-19 (Zuo et al., 2020). Furthermore, exacerbated aggregation of NET (NETs) could alter vascular districts and damage tissues (Leppkes et al., 2020). In the vascular system, NETs determine platelet activation and thrombosis, probably due to the release of histones that can be recognized through toll-like receptors (TLRs) on platelets and immune cells (Becker, 2020).

A recent report described that NET formation increases in COVID-19 patients undergoing mechanical ventilation (Zuo et al., 2020).

Patients with severe forms of COVID-19 show a marked increase in neutrophils compared to less severe subjects (Huang C. et al., 2020).

Midkine promotes the trafficking of neutrophils in myocardium and the NET formation in myocarditis (Weckbach et al., 2019). We suggest the occurrence of an important interplay between Midkine, PMN, NETs, and COVID-19. In this regard, we hypothesize that Midkine could promote neutrophil infiltration and NET formation in the myocardium *via* LRP1.

Moreover, it is likely that the Midkine-dependent promotion of neutrophil activation and NETs formation strongly degenerates the complex homeostatic mechanism of coagulation and plays a relevant role in the determinism of thrombotic events correlated to neutrophil hyperactivation (Iba and Levy, 2018). In this regard, neutrophil hyperactivation and NETs formation have been associated with ARDS in influenza pneumonitis (Narasaraju et al., 2011) and with thromboinflammatory response and intravascular thrombosis during sepsis (Iba and Levy, 2018). Finally, the molecules involved in hemostasis, as procoagulant or anticoagulant, should be deeply investigated for their potential relationship with Midkine, such as thrombin and thrombomodulin that are described to interplay each other to determine different effects on hemostasis (Rezaie, 2010) and have associated with NETs occurrence (Toh et al., 2016): Midkine could alter the balance between procoagulant and anticoagulant and could foster thromboinflammatory response and intravascular thrombosis during COVID-19 occurrence.

## Conclusion

Since December 2019, SARS-Cov2 infection has manifested broad pandemic connotations and several pathophysiological conditions that do not limit COVID-19 to abnormal pneumonia (Cevik et al., 2020; Chen et al., 2020). In this regard, severe phases of COVID-19 present a poor prognosis in those patients underlying clinical conditions such as hypertension, chronic obstructive pulmonary disease, diabetes, and/or cardiovascular disease (Harapan et al., 2020; Nikolich-Zugich et al., 2020). Indeed, such compromised patients incur a greater risk of rapid progression to ARDS, septic-type systemic shock, coagulation dysfunction, arrhythmia and heart failure, renal and/or heart failure, hepatic dysfunction, and the occurrence of secondary infection (Cevik et al., 2020; Chen et al., 2020; Harapan et al., 2020; Nikolich-Zugich et al., 2020).

In this mini review, we suggest the potential and intriguing scenario concerning the interaction between SARS-CoV2 and Midkine, in order to understand the pathophysiological mechanisms occurring in COVID-19.

We highlight a possible involvement of Midkine in the in SARS-CoV2 infection mechanisms. Indeed, Midkine could amplify S-protein RBD sulfatation sites, increasing the binding affinity of SARS-CoV2 with ACE2 receptor. In addition, the interplay between coronavirus, Midkine, HS, LRP-1, and lipid rafts could foster SARS-CoV2 internalization.

The main feature of the immune-mediated involvement in COVID-19 is characterized by neutrophil hyperactivation. In this regard, Midkine signaling could enhance neutrophil proliferation and migration. Several studies have showed that Midkine is involved in neutrophil infiltration and chemokine expression as well as in the Netosis occurrence (Figure 1). Moreover, a crucial interplay between Midkine, neutrophils, NET, and COVID-19 might occur. Severe COVID-19 correlates with exacerbated neutrophil hyperactivation and NET occurrence. Midkine could promote neutrophil infiltration and NET formation in the myocardium *via* LRP-1. In addition, Midkine could be involved in the pulmonary remodeling and fibrosis, through the collagen deposition and the Nox1, MK, Notch2, and ACE signaling pathway (Figure 1). We overviewed literature concerning Midkine-related pathway and its receptors, highlighting a common pathway with mTOR and autophagy that SARS-Cov2 could employ to elude in order to foster virus replication.

Taken in all, we hypothesize a key role of Midkine, particularly in organ dysfunction at the basis of COVID-19 pathogenesis and also propose such protein as a potential biomarker (Table 1) of pathophysiological conditions and as a key target for new potential COVID-19 therapeutical strategies by employing anti-Midkine monoclonal antibodies to be specifically prepared for clinical use in humans.

**Table 1:** Brief suggestions for studying the implication as a biomarker of Midkine in SARS-CoV2 infection and in COVID-19 patients.

Disease stages (^*^)	SARS-CoV2 detection	Midkine detection	Immune response analysis
Mild-Moderate infection (upper respiratory symptoms)	Nasopharyngeal/oropharyngeal swabs and viral RNA levels or viral antigen or anti-SARS-CoV-2 antibodies detection	ELISA (serum or urinary samples)	Basic assessment of leukocyte populations in blood (i.e., total neutrophils, total lymphocytes, and total monocytes)
Pulmonary phase (pneumonia with all its associated symptoms)	Nasopharyngeal/oropharyngeal swabs and viral RNA levels or viral antigen or anti-SARS-CoV-2 antibodies detection	ELISA (serum or urinary samples)	Interleukin-6, Interleukin-17, Interferon-γ detection. Advanced assessment of leukocyte populations in blood (i.e., total neutrophils, total monocytes, Tregs, T and B lymphocytes)
Hyperinflammation phase (with acute respiratory distress syndrome, sepsis, and kidney and other organ failures)	Nasopharyngeal/oropharyngeal swabs and viral RNA levels or viral antigen or anti-SARS-CoV-2 antibodies detection	ELISA (serum or urinary samples)	Interleukin-6, Interleukin-17, Interferon-γ detection. Advanced assessment of leukocyte populations in blood (i.e., total neutrophils, total monocytes, Tregs, T and B lymphocytes)

* The clinical classification is based on Siddiqi and Mehra (2020) and on the “Clinical management of COVID-19” guidance published by the World Health Organization (https://www.who.int/publications/i/item/clinical-management-of-covid-19).

## Author Contributions

GS and GT equally contributed, conceptualized, and wrote the manuscript. MB contributed to the manuscript reading and editing. All authors contributed to the article and approved the submitted version.

### Conflict of Interest

The authors declare that the research was conducted in the absence of any commercial or financial relationships that could be construed as a potential conflict of interest.
